# The international Genome sample resource (IGSR): A worldwide collection of genome variation incorporating the 1000 Genomes Project data

**DOI:** 10.1093/nar/gkw829

**Published:** 2016-09-15

**Authors:** Laura Clarke, Susan Fairley, Xiangqun Zheng-Bradley, Ian Streeter, Emily Perry, Ernesto Lowy, Anne-Marie Tassé, Paul Flicek

**Affiliations:** 1 European Molecular Biology Laboratory, European Bioinformatics Institute, Wellcome Genome Campus, Hinxton, Cambridge CB10 1SD, UK; 2 Public Population Project in Genomics and Society, McGill University and Genome Quebec Innovation Centre, Montreal, Quebec, Canada

## Abstract

The International Genome Sample Resource (IGSR; http://www.internationalgenome.org) expands in data type and population diversity the resources from the 1000 Genomes Project. IGSR represents the largest open collection of human variation data and provides easy access to these resources. IGSR was established in 2015 to maintain and extend the 1000 Genomes Project data, which has been widely used as a reference set of human variation and by researchers developing analysis methods. IGSR has mapped all of the 1000 Genomes sequence to the newest human reference (GRCh38), and will release updated variant calls to ensure maximal usefulness of the existing data. IGSR is collecting new structural variation data on the 1000 Genomes samples from long read sequencing and other technologies, and will collect relevant functional data into a single comprehensive resource. IGSR is extending coverage with new populations sequenced by collaborating groups. Here, we present the new data and analysis that IGSR has made available. We have also introduced a new data portal that increases discoverability of our data—previously only browseable through our FTP site—by focusing on particular samples, populations or data sets of interest.

## INTRODUCTION

The 1000 Genomes Project cataloged human genetic variation by generating and analyzing whole genome sequencing data from more than 2500 individuals across 26 populations from five continental groups (1). All 1000 Genomes data were generated from samples with broad consent for open, public release of de-identified genetic data (2). The open nature of the data has led to its widespread use for various applications, ranging from reference panels for genotype imputation to prioritizing variants for further study to serving as a test bed for methods development (3). In order to ensure the continued usability of this valuable data collection for these and other purposes, we established the International Genome Sample Resource (IGSR) in 2015 to sustain, improve and expand the resources generated by the 1000 Genomes Project.

IGSR exists for multiple reasons. We maintain the value of the 1000 Genomes data by updating the fundamental results to be consistent with the newest versions of the human genome assembly and in the light of new analysis technology. We are collecting new types of data and new data sets generated on the 1000 Genomes samples to expand the overall resource. We also add data into IGSR from new samples with similarly open consent for release of de-identified genetic data to both enlarge the existing populations and to include populations that were not represented in the original 1000 Genomes Project.

We support several different activities within IGSR including (i) data coordination and ethical review for groups collecting data on new open samples; (ii) analysis pipelines to align sequence reads and call variants; (iii) data discovery and distribution through the IGSR data portal and FTP site; and (iv) user support and training. To collect, process and distribute the IGSR data, we leverage and have extended the infrastructure created for the 1000 Genomes Project (4). In the last year, we have added new sequence data on existing 1000 Genomes samples, incorporated the initial data for samples not sequenced in the 1000 Genomes Project, and calculated and released new alignments of the entirety of the 1000 Genomes phase 3 data to the updated human reference assembly, GRCh38.

In the following sections, we describe our data structure, how to browse and access our data and the support we provide for our users.

### Data structure

Over the course of the 1000 Genomes Project, ∼500 000 data files requiring 750 TB were added to and hosted on the project FTP site in a manner and structure designed to support the needs of the 1000 Genomes Consortium. While maintaining all of the 1000 Genomes data, we have made changes to the FTP site structure to support the expanded scope of IGSR and improve the discoverability of the data. To ensure clarity as we add new data sets, we have designed a structure and nomenclature for the elements within our structure that describes the source of the data, what type of data it is and what other similar data exist (Table 1). More information about these data elements and how they can be used to filter and discover IGSR data is below and in the data portal section.

**Table 1.** IGSR Data Element definitions

Data Element	Descriptive Example
Data Collection	Study or project level grouping of data such as the 1000 Genomes Project Phase 3 or the Human Genome Structural Variation (HGSV) Consortium.
Analysis group	The experimental strategy used to generate data. Data from the same analysis group and data collection can generally be analyzed as a coherent unit. Examples include exome sequencing or high coverage whole genome Illumina sequencing. Data from different data collections can have the same analysis group.
Data Type	Specific data description and currently one of Sequence, Alignment, Variants or Other.
Population	A defined group of samples normally collected from the same geographic location and ethnic group.
Sample	An individual who donated material to a project.

#### Data collections

Data collections are large-scale sets of related data designed to be useful for answering a host of linked questions. During IGSR's first year, the number of data collections doubled (Table 2). In addition to the 1000 Genomes Project data, we have sequence data from four additional sources and are in the process of producing alignments and, eventually, variant calls using these new collections. We anticipate that further data collections will be added to the resource in the near future.

**Table 2.** IGSR Data Collections. The number of samples, number of populations and available data types in each data collection

Data Collection	Samples	Populations	Sequence	Alignment	Variants
The 1000 Genomes Project Phase 1	1092	14	Y	Y	Y
The 1000 Genomes Project Phase 3	2504	26	Y	Y	Y
The 1000 Genomes Project GRCh38	2706	26	Y	Y	
Illumina Platinum Pedigree	17	1	Y	Y	
HGSV	9	3	Y	Y	
The Gambian Genome Variation Project	394	4	Y		
Simons Diversity Project	279	130	Y		
Human Genome Diversity Project	177	52	Y		

#### Analysis groups

The analysis group concept stems from the start of phase 1 of the 1000 Genomes Project, when it became clear that a simple study structure would be inadequate to distinguish between the low coverage and exome sequencing efforts. As IGSR takes on different data types, the analysis group designation allows us to effectively sub-divide data to facilitate the discovery and use of specific types of data. This enables discovery of coherent data sets that can be processed together. Table 3 describes the existing analysis groups.

**Table 3.** Analysis groups. The description and project for each of the IGSR analysis groups

Analysis group	Description
Exome	The 1000 Genomes Project exome sequencing
High Coverage WGS	The 1000 Genomes Project high coverage sequencing
Low Coverage WGS	The 1000 Genomes Project low coverage sequencing
Strand specific RNA	HGSV strand specific Illumina RNA-Seq
3.5 kb jump	HGSV Illumina 3.5 kb jumping library sequence
HiC	HGSV HiC chromatin conformation sequencing
PCR free high	HGSV PCR free high coverage sequencing
Strand Seq	HGSV Illumina Strand Seq sequencing
SV 7 kb mate	HGSV Illumina 7 kb insert mate pair library sequencing
SV SMRT	HGSV Single Molecule Real Time Sequencing
Illumina platinum ped	Illumina Platinum Pedigree
CG	The 1000 Genomes Project Complete Genomics sequencing
Integrated calls	The 1000 Genomes Project integrated variant calls
HD genotype chip	The 1000 Genomes Project high density genotype chip results

#### Data type

The data types represent both unprocessed submitted data in the form of raw sequence data files and processed data in the form of sequence alignments to either the GRCh37 or GRCh38 human reference assembly. Variant data includes sites of variation and genotype data arising from either sequencing or array-based assays. Data that do not fit into the existing main data categories, and which currently do not exist in sufficient qualities to warrant their own type, are classified as type ‘other’.

#### Populations and samples

As our data collections expand and new populations are added, we will manage the nomenclature to ensure that the population and sample labels remain consistent and useful. As an example, the 1000 Genomes Project, HGSV and the Illumina Platinum genome data collections all contain samples sourced from the same cell line biorepository and the sample reference numbers and population names are consistent across these three collections.

Other data collections have generated new information about existing samples. We will retain information from the studies that generated data, while, where samples are included in multiple projects, making it clear that this is the case. For example, the Simons Diversity Project uses GBR samples that were also part of the 1000 Genomes Project, but it has included more precise information about the origin of the donor: some of the GBR samples in this collection are labeled as English. In cases such as this, we will use the new information to extend our descriptions, but we will also maintain the GBR label to ensure that all the data we have for these populations can be found in one place.

### Data access

We have several access methods for the IGSR data that take advantage of the data structure outlined above to organize and filter the data and which are described in detail below.

#### Data portal

**Figure 1.**
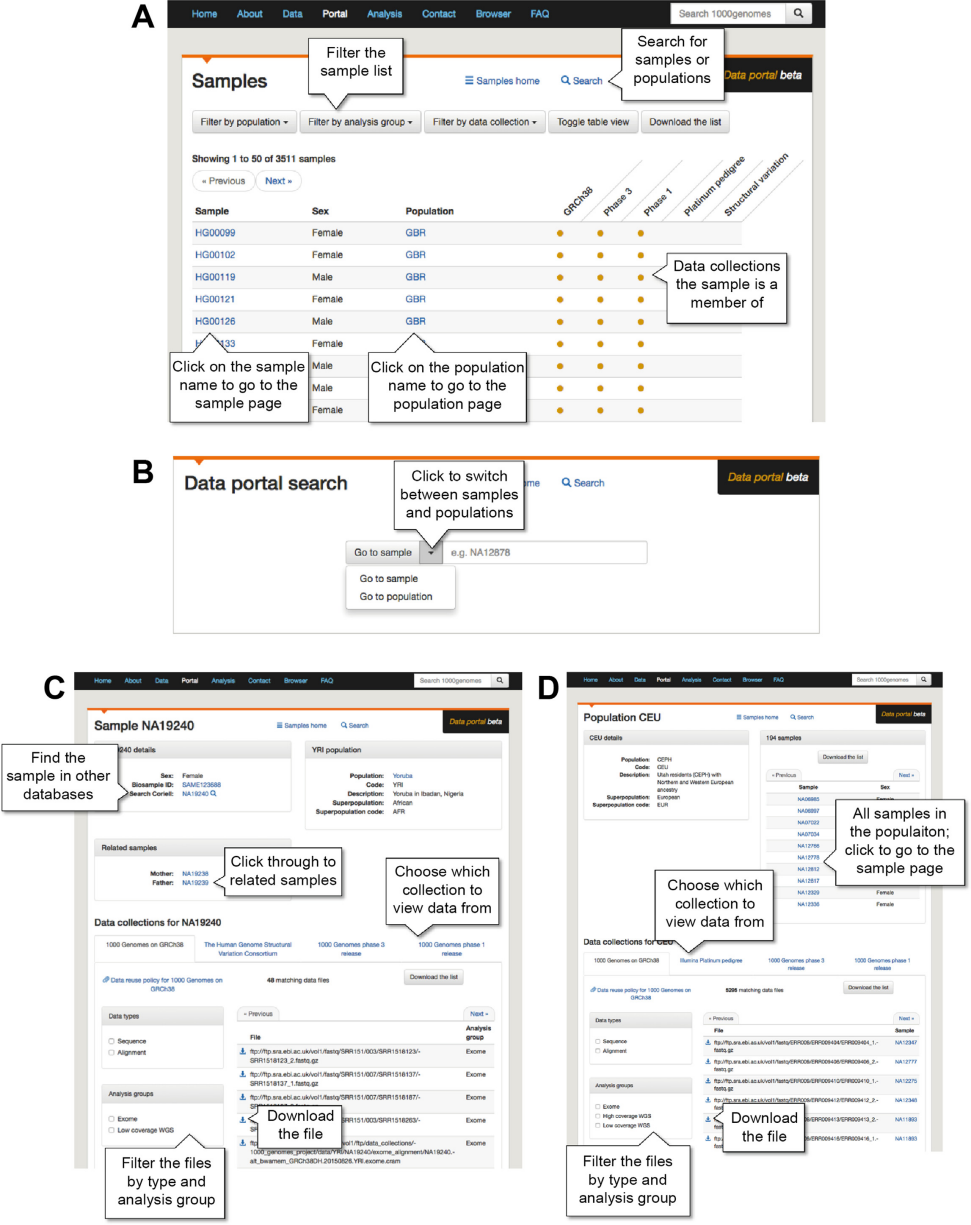
Using the IGSR Data Portal. The IGSR Data Portal is a powerful and flexible way to explore the IGSR data. (**A**) The main page of the portal is a tabular listing of all of the available samples with options for filtering by population, analysis group and data collection. Both sample reference numbers and population names are linked to further specific information and there is a link to the search interface at the top of the page. (**B**) The search page enables direct access to samples and populations. (**C**) An individual sample page gives descriptive data about a specific sample including related individuals, how to find more information about it in other databases or purchase cell lines for further experiments, if available. Sample pages also allow for filtering by data collection, data type and analysis group with the resulting data files listed on the bottom right of the page for immediate download. (**D**) Pages for each population list all of the samples with in the population as well as the options filter by data collection, data type and analysis group. As for the sample page, all available data files are linked from the list on the bottom right of the page.

We have developed a data portal to make it as easy as possible to explore the variety of data in IGSR. The portal is accessible at http://www.internationalgenome.org/data-portal/ or by clicking on *Portal* in the horizontal bar at the top of the IGSR website (Figure 1A). The default entry to the portal is a summary table listing the samples, their sex and population, and what data are available for them. This table can also be directly accessed via http://www.internationalgenome.org/data-portal/sample. Samples and data can be located using the filters at the top or by using the search to find specific samples or populations (Figure 1B). The summary entry for each sample includes links for detailed information about the sample (Figure 1C) and its corresponding population (Figure 1D), which are each described in the following paragraphs.

Clicking on a sample reference number leads to a dedicated sample page (Figure 1C). This can also be accessed by searching with the sample reference to jump straight to it. The top section of the sample page lists details regarding the sample including the population, sex and if there are any related samples also found in IGSR. Clicking on the population name brings up the population page described below. There is also a direct link to the sample in the BioSamples database (5) and to the relevant source, such as the Coriell Institute, where the lymphoblastoid cell line can be purchased. The lower section of the sample page lists all the files available for the sample on the IGSR FTP site. This list includes both files that are specific to the sample, such as FASTQ sequences and BAM alignments with their indexes, as well as files that include the sample among other data, such as VCF files containing genotypes from the sample. This section includes the data collections that include the sample and the tab for each collection lists all of the files for the given sample that are related to the selected collection. On the left are options to filter files by data type and by analysis group. On the right are the exact locations of the data files on the IGSR FTP along with direct links for download.

In addition to the sample level pages, there are population pages with all of the data files for a selected population (Figure 1D). From the sample page, these are accessible by clicking on the population link or they can be accessed directly by clicking on the population code from the portal entry page or using the search. The top section of the page gives details about the population including description, codes and superpopulation. It also lists all the samples found in the population that can be downloaded. The sample reference numbers are linked to the appropriate sample page. The lower section lists the files available for the population. These include the sample level FASTQ and BAM files (with index files), as well as the whole collection level VCFs that include the population. As with the sample page, different data collections can be selected using the tabs, and options for filtering by data types and analysis groups are on the left. The complete set of available populations is listed on http://www.internationalgenome.org/data-portal/population.

#### FTP site

The primary location of all the data collections hosted by IGSR, including the 1000 Genomes data, is the 1000 Genomes FTP site at ftp://ftp.1000genomes.ebi.ac.uk/vol1/ftp/. When the 1000 Genomes Project was active, this site hosted all project data, including raw sequence and reference data sets, alignments, and preliminary and integrated variant calls. We now point to the primary sequence data in the European Nucleotide Archive (ENA) (6), while the FTP site hosts all the downstream analysis results and reference information. As noted above, the current FTP site is organised to manage the expanding range of data collections and data types being hosted. For example, each data collection has its own directory under ftp://ftp.1000genomes.ebi.ac.uk/vol1/ftp/data_collections, which contains README files giving details about the data collection and its data reuse statement. All other files associated with the project are also located here with sample level files, organized by population and sample, in the data directory and integrated results files generally in working or release directories for preliminary or final analysis results, respectively.

In order to support mirroring of the IGSR FTP site, we provide both a complete index of the current FTP site structure (ftp://ftp.1000genomes.ebi.ac.uk/vol1/ftp/current.tree), which lists the location of every file; its md5 checksum and its last updated timestamp; and a series of changelog files that list any new, removed or renamed files. Data can be downloaded from our site via FTP and HTTP protocols. We also make the data available via two fast download protocols, Aspera and Globus GRID FTP, to support large volume downloads.

#### Browsing IGSR's data

Project specific genome browsers were created and regularly updated for each phase of the 1000 Genomes Project using the Ensembl infrastructure (7). We maintain browsers associated with the final data set from each of three 1000 Genomes publications: http://pilotbrowser.1000genomes.org (8), http://phase1browser.1000genomes.org (9) and http://phase3browser.1000genomes.org (1), but we will not create any future dedicated browser deployments. The existing browsers will continue to be maintained in their current location but, in order to take advantage of the new features and data that Ensembl continues to add, we now recommend users visualize our data through the main Ensembl website, ensuring they get the best experience when browsing our data. Ensembl has established and continues to update a version of their browser on the GRCh37 human reference (http://grch37.ensembl.org/index.html), which contains all the Phase 3 1000 Genomes Project data and is updated with new data and new tools. The main Ensembl browser on the GRCh38 assembly also contains all the existing 1000 Genomes variant calls and we will rapidly add new integrated call sets to the browser, initially using the TrackHub protocol (10) and with a full import to Ensembl after the data are archived in dbSNP. We are working with the Ensembl team to move the tools that were previously only available in the 1000 Genomes Browser, such as the Allele Frequency Calculator and VCF to PED converter, to the Ensembl tools infrastructure.

### IGSR website

The IGSR website (http://www.internationalgenome.org) provides summary information, project descriptions, data reuse policies and announcements about the project. It also includes training materials and an extensive Frequently Asked Questions (FAQ) section. This content is designed to provide context about the project and the data we hold and to facilitate discovery and use of the data. All announcements are also made on our Twitter feed (@1000genomes).

### Data reuse policies

The 1000 Genomes Project released all of its data prior to publication with a data use statement following Fort Lauderdale and Toronto principles (11) to allow broad use, but restricting first publication of global analysis to the Consortium. As the project data have now all been published, it is completely free of even these restrictions. IGSR data come from many different sources, including published and unpublished projects. Although for data to be included in IGSR, we insist on policies that permit pre-publication data reuse for unpublished data, each project creates a specific data use policy. We ensure a particular data collection's policy is stated clearly on the FTP site and the website (e.g ftp://ftp.1000genomes.ebi.ac.uk/vol1/ftp/data_collections/hgsv_sv_discovery/README_hgsvc_datareuse_statement.md) and recommend that any questions about appropriate usage be directed to info@10000genomes.org.

### User support

To ensure that everyone wanting to take full advantage of IGSR's rich data and tools is able to do so, we provide several types of support. As well as the data portal and FAQ, we also have a dedicated email helpdesk (info@1000genomes.org), where users can ask questions about our data collections and tools. We also provide training workshops in collaboration with the Ensembl outreach activities to deliver courses about the 1000 Genomes Project, the broader IGSR data collections and/or Ensembl at a user's home institution.

### Future work

The goal of the International Genome Sample Resource is to continue to grow what is already the world's largest open collection of human variation data. We are actively seeking and are funded to support new collaborative partners to expand our population coverage and increase the variety of data that we hold on samples that have whole genomes already available. As we increase the data collection, we will develop our infrastructure to ensure all users can access and make use of the available data. We will also improve the data portal interface to ensuring IGSR remains the valuable resource it is today.
